# Cycling and heart failure: A 2-sample Mendelian randomization

**DOI:** 10.1097/MD.0000000000037619

**Published:** 2024-03-29

**Authors:** Jianwei Zhou

**Affiliations:** a People Hospital of Xishuangbanna Dai Autonomous Prefecture, Jinghong, Yunnan, China.

**Keywords:** cycling, heart failure, mendelian randomization

## Abstract

Heart failure (HF) is a major cause of mortality worldwide. Cycling, an aerobic exercise, is believed to have a more effective rehabilitative impact on patients with heart failure. Previous studies have demonstrated the benefits of exercise in patients with HF. However, a precise causal relationship remains unknown. Two-sample Mendelian randomization (MR) was used to investigate the potential causal relationship between regular cardiac cycling and heart failure (HF) development. Data from the IEU OpenGWAS project, an extensive genetic study involving a diverse group of European males and females was used to determine how choices related to physical activity, such as cycling, impact cardiovascular well-being. To ensure reliability and robustness, the MR-Egger regression, weighted median, and random effects with inverse variance weighting methods were used. The key findings were summarized using odds ratio (OR) and 95% confidence intervals (CI). The MR-Egger, weighted mean, and inverse variance weighted (IVW) estimated superiority ratios were 0.960 (95% CI: 0.909–1.013), 0.985 (95% CI: 0.962–1.009), and 0.982 (95% CI: 0.966–0.998), respectively, indicating a significant association between cycling and a decreased risk of heart failure. These findings suggest that cycling, a form of moderate and easily accessible physical activity, may be a protective factor against HF. These findings correlate with those of previous studies regarding the crucial role of regular physical activity for the prevention and management of cardiovascular disease. The outcomes of this MR analysis can be used in the development of public health policies and aid individuals making lifestyle choices that promote heart health.

## 1. Introduction

Heart failure (HF) occurs when the heart cannot sufficiently circulate blood or provide adequate oxygen to meet metabolic demands.^[1]^ There are 65 million reported cases of heart failure worldwide, and this number is projected to increase in correlation with the aging global population.^[2]^ HF poses a significant burden on public health with substantial costs owing to the high risk of morbidity and mortality.^[3]^ HF treatment is primarily supportive and includes standard heart failure medications, mechanical aids, and heart transplantation; although medications and surgery can alleviate the symptoms of HF, these approaches do not completely cure HF,^[4]^ non-pharmacologic treatments to alleviate the symptoms of HF must be identified.

**Figure 1.**
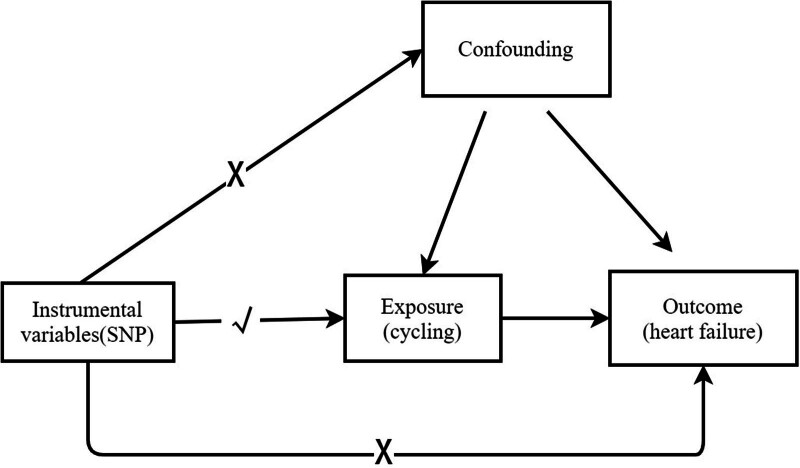
Schematic representation of Mendelian randomization analysis. SNP = single-nucleotide polymorphism.

Exercise is considered an effective non-pharmacological therapy for HF. Engaging in physical activity is effiective for the primary and secondary prevention of cardiovascular disease.^[5]^ In a previous meta-analysis, exercise was found to be effective in improving survival and hospitalization rates in patients with HF.^[6,7]^ Riding a bicycle reduces traffic congestion and improves air quality. Cycling also reduces the risk of cardiovascular disease,^[8]^ and is considered an effective aerobic exercise. To further investigate the causality and strength of the association between cycling and HF, Mendelian randomization (MR) was performed. Genetic variations, including single nucleotide polymorphisms (SNPs), can function as instrumental variables (IVs) that altering disease risk factors or exposures. The utilization of MR investigations can augment the inferential process related to the causal connections between exposure and outcome (Fig. 1).^[9]^ In accordance with Mendel inheritance principles, genetic variations remain uninfluenced by confounding factors owing to their random allocation during gamete formation.^[10]^

## 2. Data and methods

### 2.1. Data sources

Genome-wide association study (GWAS) data for cycling and HF were obtained from the IEU OpenGWAS project (https://gwas.mrcieu.ac.uk). All the datasets used in this study included male and female individuals of European origin. The cycling database (ukb-a-481) contained 10,894,596 SNPs with a sample size of 335,650 individuals, and the HF database (ukb-d-HEARTFAIL) contained 9858,439 SNPs (Table 1). This study was a reevaluation of existing and publicly available data; therefore, no ethical authorization was necessary.

**Table 1:** Information of the GWAS database in the 2-sample mendelian randomization.

Trait	ID	Sample size	SNP	Population	Sex
Types of transport used (excluding work): Cycle	ukb-a-481	335,650	10,894,596	European	Males and females
Heart failure	ukb-d-HEARTFAIL	361,194	9858,439	European	Males and females

GWAS = genome-wide association study, SNP = sigle-nucleotide polymorphism.

### 2.2. Data processing

Typically, a threshold of *P* < 5.0 × 10^−8^ was deemed significant to confirm that the SNPs were associated with cycling; however, no SNPs met this criterion during screening. Therefore, the threshold was changed to *P* < 5.0 × 10^−6^. The TwoSample MR package of R software (R Development Core Team, Global Collaboration) was used to exclude the interference of chain imbalance, and the parameters were set at *P* < 5.0 × 10^−6^, *r*^2^ = 0.001, and kb = 10000. IVs with an F-value > 10 were excluded from the analysis. In addition, potential confounding factors were carefully eliminated to identify SNPs associated with cycling.^[11]^

### 2.3. MR analysis

The Wald ratio technique was used to evaluate the impact of IVs on causal estimates. The inverse variance weighted (IVW) test was used to calculate the causal effect values in the absence of horizontal pleiotropy to ensure unbiased estimates. Based on the presence or absence of heterogeneity, a fixed- or random-effects model was used with the IVW method. The effect size is indicated by the odds ratio (OR) and corresponding 95% confidence interval (CI). The weighted median method^[12]^ and the MR-Egger test^[13]^ were also used as supplementary approaches.

### 2.4. Sensitivity analysis

Several analytical methods were used to conduct sensitivity analysis. Cochran Q test was used to evaluate heterogeneity among the individual SNPs, with significant values indicating significant heterogeneity. The leave-one-out method was used to assess the sensitivity of the findings. When the exclusion of an SNP yields a *P* value > .05, the SNP affects the results.^[14]^

## 3. Results

**Figure 2.**
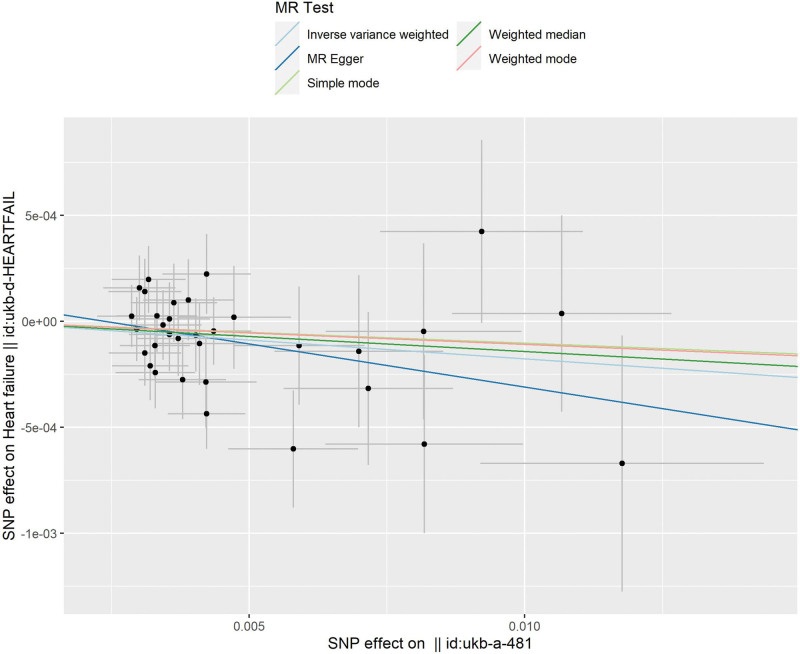
Scatter plot of cycling and heart failure.

**Figure 3.**
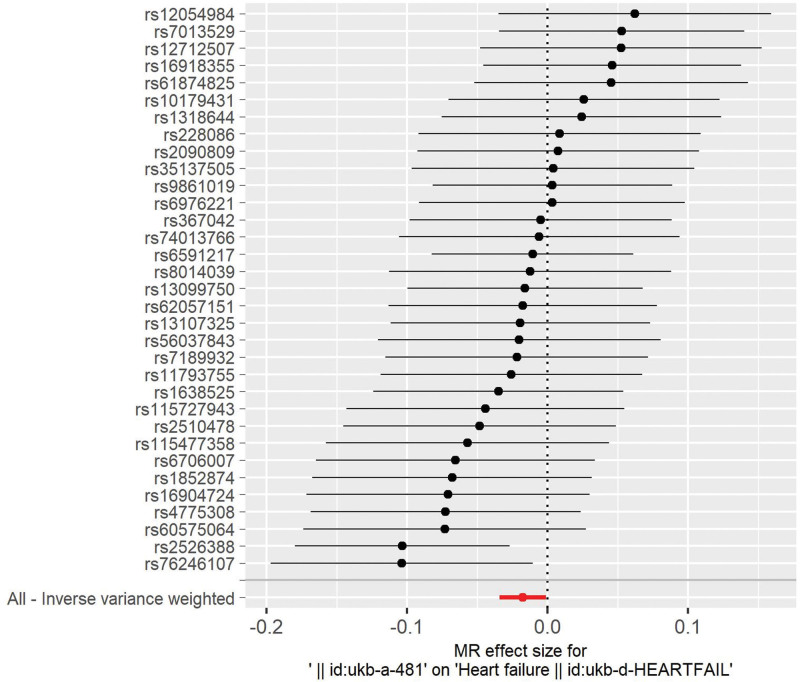
Forest plot of cycling and heart failure.

**Figure 4.**
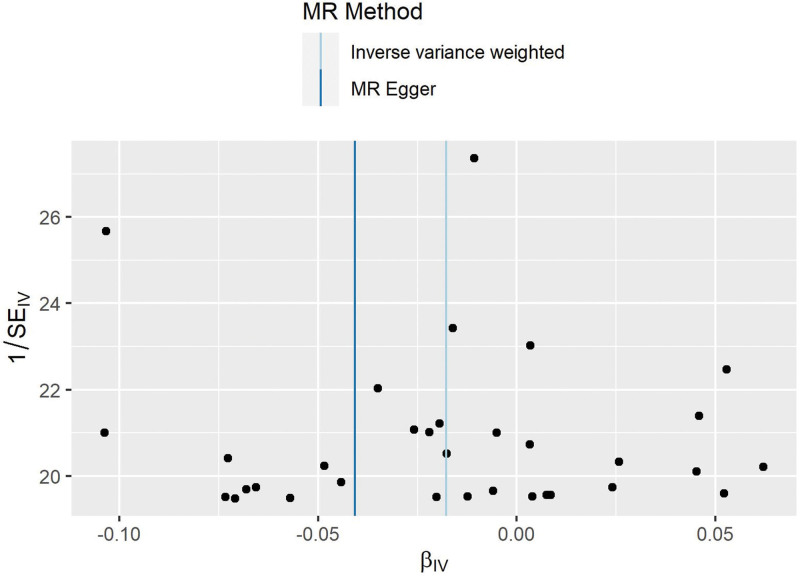
Funnel plot of cycling and heart failure.

**Figure 5.**
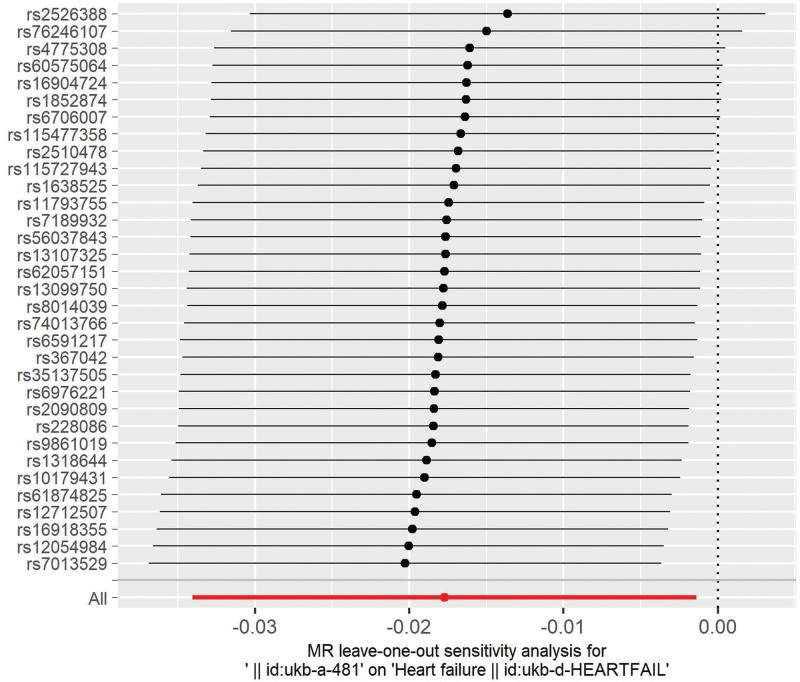
Analysis of cycling and heart failure by the leave-one-out method.

Thirty-three SNPs were retained for this study (Table 2). The causal relationship between cycling and HF was found to be associated with HF using the MR-Egger (OR: 0.960, 95% CI: 0.909–1.013), weighted mean (OR: 0.985, 95% CI: 0.962–1.009), and IVW (OR: 0.982, 95% CI: 0.966–0.998) methods (Table 3). Cycling has been shown to reduce the risk of developing HF (Figs. 2 and 3). No heterogeneity was identified using the MR Egger (*P* = .65) or IVW (*P* = .66) methods (Fig. 4). No SNP were identified as efficacious in the leave-one-out analysis (Fig. 5).

**Table 2:** Features of the SNPs linked to cycling and their connections with heart failure.

SNP	EA	OA	chr	Exposure			Outcome		
				β	SE	*P*	β	SE	*P*
rs10179431	C	T	2	0.0039	0.000819	1.95E-06	0.0001	0.000192	.600371
rs115477358	G	A	2	−0.01177	0.002573	4.79E-06	0.000671	0.000604	.266459
rs115727943	T	C	6	−0.00716	0.001538	3.22E-06	0.000317	0.000361	.37978
rs11793755	A	G	9	−0.0041	0.00083	7.93E-07	0.000106	0.000195	.586619
rs12054984	A	T	5	0.003179	0.000672	2.28E-06	0.000197	0.000157	.21001
rs12712507	G	T	2	−0.00301	0.000657	4.49E-06	−0.00016	0.000154	.306709
rs13099750	A	T	3	0.004034	0.000736	4.16E-08	-6.49E-05	0.000172	.706131
rs13107325	T	C	4	−0.00591	0.00119	6.83E-07	0.000115	0.000278	.680522
rs1318644	G	A	10	0.003635	0.000786	3.80E-06	8.75E-05	0.000184	.63456
rs1638525	C	G	17	−0.00329	0.000638	2.45E-07	0.000115	0.000149	.440462
rs16904724	A	C	8	0.008179	0.001791	4.97E-06	−0.00058	0.00042	.167617
rs16918355	C	T	9	0.009219	0.001839	5.35E-07	0.000423	0.000431	.32609
rs1852874	A	G	12	0.004219	0.000916	4.06E-06	−0.00029	0.000214	.180506
rs2090809	C	T	6	−0.00333	0.000727	4.62E-06	−2.48E-05	0.00017	.884002
rs228086	A	G	21	0.002872	0.000626	4.55E-06	2.44E-05	0.000147	.86807
rs2510478	A	G	11	0.003111	0.000656	2.13E-06	−0.00015	0.000154	.327686
rs2526388	C	T	3	0.004227	0.000703	1.86E-09	−0.00044	0.000165	.00799
rs35137505	T	G	1	0.004728	0.001034	4.79E-06	1.82E-05	0.000242	.940028
rs367042	A	T	21	−0.00344	0.000699	8.65E-07	1.73E-05	0.000164	.915849
rs4775308	C	A	15	−0.00379	0.000793	1.77E-06	0.000276	0.000186	.137948
rs56037843	C	T	4	0.006991	0.001531	4.93E-06	−0.00014	0.000358	.693621
rs60575064	T	C	19	−0.0033	0.000722	4.94E-06	0.000242	0.000169	.152763
rs61874825	C	G	10	−0.00311	0.000661	2.53E-06	−0.00014	0.000155	.363564
rs62057151	T	C	17	0.003558	0.000739	1.48E-06	-6.26E-05	0.000173	.717961
rs6591217	A	G	11	−0.00436	0.00068	1.45E-10	4.68E-05	0.000159	.769113
rs6706007	G	A	2	0.003206	0.000693	3.71E-06	−0.00021	0.000162	.195338
rs6976221	G	T	7	0.003555	0.000732	1.21E-06	1.15E-05	0.000172	.946776
rs7013529	T	C	8	0.004229	0.000804	1.44E-07	0.000223	0.000188	.235267
rs7189932	G	A	16	−0.00372	0.000756	8.76E-07	8.16E-05	0.000177	.644683
rs74013766	T	G	15	−0.00817	0.001776	4.25E-06	4.84E-05	0.000415	.907172
rs76246107	A	G	19	−0.0058	0.001179	8.56E-07	0.000602	0.000276	.029275
rs8014039	G	T	14	0.002963	0.000648	4.87E-06	-3.67E-05	0.000152	.809005
rs9861019	C	A	3	0.010672	0.00199	8.14E-08	3.66E-05	0.000464	.937038

SNP = single nucleotide polymorphism.

**Table 3:** MR regression results of the 3 methods.

method	β	SE	*P*val	OR (95%CI)
MR Egger	−0.04069	0.027754	.152736	0.960 (0.909–1.013)
Weighted median	−0.01421	0.012263	.246649	0.985 (0962–1.009)
Inverse variance weighted	−0.01771	0.008323	.033304	0.982 (0.966–0.998)

CI = confidence interval, MR = mendelian randomization.

## 4. Discussion

Cycling is an important aerobic exercise beneficial for the prevention and management of HF. However, it remains unclear whether the association between cycling and HF is causal. This study investigates the causal relationship between cycling and HF using 2-sample MR. The results of this study indicate that cycling has a positive effect on HF, suggesting that cycling can be beneficial for the prevention and management of HF.

Cycling to work lowers the risk of clinically significant cardiovascular risk factors including obesity, hypertension, and hyperlipidemia.^[8]^ Among Danish people aged 50 to 65 years, those who regularly cycled as leisure or a means of commuting were 11% to 18% less likely to have a heart attack, and as little as 30 min of cycling per week could provide some protection for the heart.^[15]^ Treadmill and bicycle-based exercises are effective and safe for patients with HF, as they reduce all-cause mortality and rehospitalization rates by 11%, reduce cardiovascular death and rehospitalization rates by 15%, and significantly improve exercise endurance with a favorable safety profile.^[16]^

The development of HF involves myocardial remodeling, which is primarily linked to myocardial hypertrophy, myocardial fibrosis, and apoptosis.^[17]^ Cardiac diastolic function improvement is achievable in mouse models of HF through aerobic exercise. The main underlying mechanism involves shielding of the heart and diminishing myocardial oxidative stress via the β3-AR-nNOS-NO pathway. Aerobic exercise may increase the expression of β3-adrenergic receptors, activating neuronal nitric oxide synthase and resulting in the generation of nitric oxide (NO).^[18]^ NO plays an important role in regulating myocardial contractility,^[19]^ reducing myocardial damage, and improving cardiac function. Physical activity impedes ventricular remodeling by suppressing TGF-β overexpression and adjusting the dynamic equilibrium between MMP-1 and TIMP-1. This intervention decreased collagen accumulation and myocardial fibrosis.^[20]^ Regular physical activity improves HF outcomes by suppressing uncoupling protein 2 expression. This is achieved by activating the irisin/reactive oxygen species/uncoupling protein 2 pathway, which establishes a novel equilibrium for myocardial mitochondrial oxidative stress.^[21]^ Therefore, physical activity can reduce damage to the heart muscles and discourage cell death via these interconnected routes, which reduces the extent of cardiac restructuring, enhances heart performance, and slows the advancement of cardiac insufficiency.

This study has some limitations. The data used in this study were from individuals of European descent, which limits the generalizability of the results. Furthermore, owing to insufficiently detailed clinical data, subgroup examinations were not possible, which impedes the identification of precise causal relationships.

## 5. Conclusion

Cycling and HF were negatively associated in this MR study, highlighting the importance of promoting the benefits of cycling for HF prevention. Cycling is a cost-effective and environment-friendly exercise, making it an attractive activity to incorporate into patients’ lifestyles. It is important to increase public awareness regarding the advantages of cycling in the prevention of HF. In future research endeavors, it is imperative to conduct long-term prospective cohort studies that meticulously track participation in cycling activities, along with subsequent alterations in the risk of heart failure across diverse demographic groups, including age, sex, and health status. These studies are essential to elucidate the longitudinal impact of cycling on the incidence and progression of heart failure. This approach will not only enhance our understanding of the relationship between physical activity and cardiovascular health but also aid in formulating more precise public health guidelines and interventions.

## Acknowledgments

All genetic summary data were obtained from the IEU OpenGWAS project. We thank all participants and investigators for contributing to the GWAS data.

## Author contributions

**Data curation:** Jianwei Zhou.

**Formal analysis:** Jianwei Zhou.

**Funding acquisition:** Jianwei Zhou.

**Investigation:** Jianwei Zhou.

**Methodology:** Jianwei Zhou.

**Project administration:** Jianwei Zhou.

**Resources:** Jianwei Zhou.

**Software:** Jianwei Zhou.

**Supervision:** Jianwei Zhou.

**Validation:** Jianwei Zhou.

**Visualization:** Jianwei Zhou.

**Writing – original draft:** Jianwei Zhou.
